# The genetic basis of colonic adenomatous polyposis syndromes

**DOI:** 10.1186/s13053-017-0065-x

**Published:** 2017-03-16

**Authors:** Bente A. Talseth-Palmer

**Affiliations:** 1 Department of Laboratory Medicine, Children’s and Women’s Health, Faculty of Medicine and Health Sciences, Norwegian University of Science and Technology, Trondheim, 7491 Norway; 2 Clinic for Medicine, Møre og Romsdal Hospital Trust, Molde, Norway; 3 School of Biomedical Sciences and Pharmacy, Faculty of Health and Medicine, University of Newcastle, Newcastle, NSW Australia; 4 Hunter Medical Research Institute, Newcastle, NSW Australia; 5 Clinic for Medicine, Library, Molde Hospital, Parkvegen 84, Molde, 6407 Norway

**Keywords:** Genetics, FAP, Genotype-phenotype, Modifier genes, MAP, NAP, PPAP

## Abstract

Colorectal cancer (CRC) is one of the most common forms of cancer worldwide and familial adenomatous polyposis (FAP) accounts for approximately 1% of all CRCs. Adenomatous polyposis syndromes can be divided into; familial adenomatous polyposis (FAP) – classic FAP and attenuated familial adenomatous polyposis (AFAP), MUTYH-associated polyposis (MAP), NTHL1-associated polyposis (NAP) and polymerase proofreading-associated polyposis (PPAP). The polyposis syndromes genetics and clinical manifestation of disease varies and cases with clinical diagnosis of FAP might molecularly show a different diagnosis.

This review examines different aspects of the adenomatous polyposis syndromes genetics and clinical manifestation of disease; in addition the genotype-phenotype and modifier alleles of FAP will be discussed. New technology has made it possible to diagnose some of the *APC* mutation negative patients into their respective syndromes. There still remain many molecularly undiagnosed adenomatous polyposis patients indicating that there remain causative genes to be discovered and with today’s technology these are expected to be identified in the near future. The knowledge about the role of modifier alleles in FAP will contribute to improved pre-symptomatic diagnosis and treatment.

New novel mutations will continually be discovered in genes already associated with disease and new genes will be discovered that are associated with adenomatous polyposis. The search for modifier alleles in FAP should be made a priority.

## Background

Colorectal cancer (CRC) is one of the most common form of cancer worldwide [1]. CRC development is considered to be a result of a combination of genetic and environmental factors and it is estimated that up to 35% of all CRCs are associated with a genetic predisposition [2]. Familial adenomatous polyposis (FAP) accounts for approximately 1% of all CRCs [3] and is an autosomal dominantly inherited condition where affected individuals develop hundreds to thousands of adenomas (polyposis) throughout the colon and rectum at unusually young ages. The disease is due to mutations in the *adenomatous polyposis coli (APC)* gene. If left untreated (if the colon is not removed), one or more of these adenomas invariably become malignant with almost 100% penetrance. Although prophylactic surgery significantly reduces the mortality associated with FAP, extra-colonic manifestations of the disease are now more clinically relevant, most notably desmoid tumours, which are hard to treat and a major cause of death [4, 5]. Attenuated FAP (AFAP) is a milder form of classic FAP with less polyps (<100) and a later age of polyp/cancer onset.

Herein an update on the genetic basis of FAP and other adenomatous polyposis syndromes (MAP, NAP and PPAP) is discussed. This review focuses on the genetics of FAP, the genetic phenotypes of the disease (genotype-phenotype correlations) and studies on modifier alleles. It also gives an update on other adenomatous polyposis syndromes. Recent findings are highlighted and gaps are identified in current literature, and consideration is give as to how these may be addressed through genomic approaches. A summary of the different adenomatous polyposis syndromes is shown in Table 1.

**Table 1:** Summary of adenomatous polyposis syndrome genetics, inheritance and clinical manifestation

Name	Abbreviation	Genetics	Inheritance	Clinical manifestation
Classical familial adenomatous polyposis	FAP	Germline *APC* mutations	Autosomal dominant	100–1000s colorectal polyps which manifests at age; early childhood-mid 30s (typically 16) and rapidly increasing. Almost 100% risk of CRC if left untreated. Treatment recommendations, colectomy after adenomas emerges. Associated with adenomatous polyps colon, CRC, fundic gland polyps, adenomatous polyps in the duodenum and periampullary region, extra intestinal lesions (fibromas, lipomas, sebaceous and epidermoid cysts = Gardner syndrome), desmoid tumours (benign soft-tissue tumours), congenital hypertrophy of the retinal pigment epithelium (CHRPE), and cancers of the brain (medulloblastoma = Turcot syndrome), pancreas, thyroid, gall bladder, bile duct and adrenal gland.
Attenuated familial adenomatous polyposis	AFAP	Germline *APC* mutations	Autosomal dominant	<100 colorectal polyps (typically 30) at age typically between 40 and 70 years (average 55). Estimated 70% CRC risk by age 80 years. Treatment recommendations, colectomy may be necessary but for some polyps are limited enough in number that surveillance of colon is sufficient. Associated with adenomatous polyps of the colon, CRC, upper gastrointestinal polyps, duodenal and gastric adenomas and fundic glad polyps. In addition, hepatoblastoma, gastric and breast adenocarcinoma have been documented.
MUTYH associated polyposis	MAP	Germline biallelic *MUTYH* mutations	Autosomal recessive	Usually < 100 polyps at average age of mid-50s and give a high risk of CRC. Associated with malignancies of the duodenum, ovary, bladder and skin.
NTHL1 associated polyposis	NAP	Germline homozygous or compound heterozygous *NTHL1* mutations	Autosomal recessive	Polyp number unknown as it is a recently discovered association but an extended spectrum of cancer diagnosis has been observed (CRC, endometrium, duodenum, skin, breast, pancreatic and others). Multiple primary tumours in all patients.
Polymerase proofreading associated polyposis	PPAP	Germline *POLE* or *POLD1* mutations	Autosomal dominant	Polyp number unknown, also recently discovered. Associated with multi-tumour phenotypes like colon/pancreas/ovaries/small intestine and colon/ovarian/endometrial/brain.

## Familial Adenomatous Polyposis (FAP) genetics

FAP is a result of germline mutations in *APC* [6, 7]. *APC* is a tumour suppressor gene that plays a central role in the Wnt signalling pathway. A detailed review of *APC* structure and function has been published by Half et al. [8]. In brief, *APC* is located on locus 5q21-22, consists of 15 coding exons (number of exons have increased to 18 after the identification of two promoter regions of *APC* [9]) and is 8532 bp in size which translates to a protein comprising 2843 amino acids [10]. Somatic mutations in *APC* is also a key molecular event in sporadic colorectal cancer present in about 80% of patients [10]. Two codons (1061 and 1309) are mutational hot-spots and account for 11 and 17% of all germline mutations, respectively [11] and are common sites of somatic change in sporadic CRC. But in a number of patients no underlying germline mutation can be identified [12, 13].

*APC* plays a central role in the Wnt-signalling pathway, especially in regards to the degradation of β-catenin within the cell cytoplasm. If *APC* is mutated, the β-catenin-Tcf complex is not suppressed and leads to constitutive activation of several genes and oncogenes controlling cell growth and division [10]. Mutations in *APC* affect the ability of the cell to maintain normal growth and function, which results in cell overgrowth/adenoma formation.

About 25% of people with FAP do not have any family history of disease and harbour a de novo mutation in *APC* without any clinical or genetic evidence of FAP in the family [14–16]. One study suggests that a 5 bp deletion of codon 1309 (c.3927_3931del) is over-represented in patients with a suspected de novo mutations (29%) and in proven de novo mutation carriers (45%) [17], supporting the view of codon 1309 as a hot-spot for mutations.

New methods that can screen genomic loci at great depths are revealing that patients that were thought to be *APC* mutation negative have pathogenic germline heterozygous *APC* mutations [18], *APC* promoter mutations [9], deep intronic mutations [19], complex genomic rearrangements [20], somatic mutations or *APC* mutation mosaicism [12, 21–23].

### Classic FAP

Classic FAP (OMIM #175100) refers to patients who are diagnosed with FAP due to the development of more than 100 adenomatous colorectal polyps from early childhood (typically at age 16) who harbour an *APC* germline mutation. On average, cancer develops a decade after the appearance of adenomas and if the colon is left untreated most patients develop CRC by 40 years of age [8]. Other gastrointestinal manifestations include fundic gland polyps (which occurs in approximately 90% of FAP patients and are mostly benign [24]), adenomatous polyps in the duodenum and periampullary region (lifetime risk has been reported to reach 100% [25, 26]), and small bowel adenomas [8]. Extra-colonic manifestations are common but rarely malignant and include [8]; desmoid tumours (benign soft-tissue tumours that can be fatal due to progressive invasion into surrounding tissues [5, 27]), cutaneous lesions such as fibromas, lipomas, sebaceous and epidermoid cysts (present in Gardner syndrome [28], a phenotypic variant of FAP), congenital hypertrophy of the retinal pigment epithelium (CHRPE, which is a lesion causing discoloration in the ocular fundus – low-grade adenocarcinoma has been described in these lesions [29]), brain tumours (mainly medulloblastoma, described in Turcot’s syndrome, another phenotypic variant of FAP), hepatoblastoma, dental abnormalities, cancer of the pancreas, thyroid, gallbladder, bile duct and adrenal glands [8, 30–32].

### Attenuated familial adenomatous polyposis (AFAP)

AFAP is a phenotypic variant of FAP; patients develop less than 100 polyps, delayed polyp growth and later age of cancer onset. Germline *APC* mutations are also present in these patients, which are mainly observed in three sections of the gene (first 5 exons, exon 9 and in the distal 3′end of *APC*) [33]. Mean age of polyp diagnosis in AFAP patients is variable but on average in fourth to fifth decade of life, with cancer developing 10–15 years later [8, 34]. Screening is suggested to start late teens to mid-20s [34]. As with FAP the most common extra-colonic manifestations are upper gastrointestinal polyps, duodenal and gastric adenomas and fundic gland polyps [33]. Extra-colonic manifestations in AFAP are rare but hepatoblastoma, gastric and breast adenocarcinoma have been documented [8, 33].

### FAP genotype-phenotype

In families with FAP, considerable variability in disease expression is observed within and between families harbouring identical *APC* mutations [13, 35–37] and it has been shown that the greater the number of colorectal adenomas, the greater the risk of CRC [38]. It has been demonstrated that there is significant variation with respect to age of onset of intestinal symptoms and the development of CRC, even in patients with the same mutations [13]. Haplotype reconstruction from pedigrees have revealed there is no evidence for a specific *APC* haplotype associated with disease severity [39]. Genotype-phenotype correlations have been associated with the location of germline mutations within *APC* that are related to disease severity and the expression of extra-colonic disease [40–42], see figures in Half et al. [8] and Macrae [10]. Patients with mutations in the mutation cluster region (MCR), located between codons 1286 and 1513 [43], have generally a worse prognosis with earlier disease onset than those with mutations outside this region [44]. Germline mutations at codon 1309 is associated with most severe disease [45], while milder forms with less than 100 adenomas and later ages of onset (AFAP) are associated with codons <157, 312–412 and >1595 [33, 41]. CHRPE has been associated with mutations between codons 457 and 1444 and susceptibility to desmoid tumours is correlated with mutations between codons 1395 and 2000, with slight variability in codon ranges between reports [8, 10, 46]. There is evidence of large phenotypic variation among patients with identical germline mutations [13], strongly suggesting the existence of FAP modifier alleles.

### FAP modifier alleles

The phenotypic variation of *APC* ^*Min*^ mouse model of FAP reveal among different inbred strains the importance of modifier alleles [47]. There is evidence to suggest that these phenotypic differences are caused by segregating modifier alleles that impact adenoma number [47]. Several have been found in the *min* mouse model. The best known modifier is possibly *Mom1* (modifier of Min 1), which is semi-dominant - each copy affects tumour multiplicity by a factor of approximately 2 [47]. *Pla2g2a* (found at the same region as Mom1) has also been shown to affect the net growth rate of adjacent tumours [48]. The exact mechanism by which it influences tumorigenesis remains unresolved [47] and the effort of linking *PLA2G2A* to FAP in humans has failed [49, 50], illustrating the difficult task of searching for modifier alleles in FAP. Many different genetic modifiers of the *Apc* knockout mouse models have been found, affecting karyotypic stability, DNA mutation rate, recombination rates, differentiation, DNA methylation, stromal regulation, cell growth and proliferation (reviewed in [47, 51]). There are claims that mouse models are essential in identifying modifiers of human disease and by using an Apc (Min/+) model have identified seven genes that are the most likely candidates for the Mom5 modifier [52]. Recently it has been reported that a new *Xenopus* tumour model might be especially useful for identifying or characterising modifier genes associated with *APC* mediated tumour formation [53].

Genome wide association studies (GWASs) have identified approximately 40 CRC susceptibility loci, where each loci gives a small increased risk of CRC [54]. The risk associated with each variant is too small on their own for translation to testing in clinical practice but the development of algorithms estimating cumulative risk are expected to lead to clinical application [54]. Two of these SNPs have been associated with Lynch syndrome [55, 56] and recently the same SNPs (rs16892766 and rs3802842) have been associated with adenoma number in *APC* mutation carriers causing a more sever FAP phenotype [57].

Several studies suggest that low-penetrant susceptibility genes may play an important role in the development of sporadic CRC [58–63]. There is evidence to show that the variation in FAP severity (which have been shown to be independent of *APC* mutations and most likely the action of modifier alleles), is expected to result in different rates of adenoma number rather than differences in tumour progression [64]. Modifier genes can influence individual susceptibility to cancer by enhancing or suppressing disease initiation, growth and/or progression. The pattern of intra-familial variation in colonic FAP severity is consistent with the action of modifier genes [39, 64–66]. As described above there is plenty of evidence from animal models for the existence of FAP modifiers and knowledge of modifier genes will contribute to better prophylactic measures for FAP patients [67]. It is important that the search for modifier genes/alleles continues.

## Other adenomatous polyposis syndromes

Some of the recently discovered adenomatous polyposis syndromes are recessively inherited and present a diagnostic challenge. Individually, the other polyposis syndromes are very rare and may show overlapping phenotypes.

### MUTYH-associated polyposis (MAP)

MAP (OMIM #608456) is an autosomal recessive disease caused by biallelic mutations in the base excision repair gene *MUTYH. MUTYH* is involved in base excision repair and is necessary in the amelioration of reactive oxygen species DNA damage prior to cell division [68]. In a recent study, 23% of *APC* mutation negative samples (FAP samples screened for APC mutations) were found to harbour pathogenic mutations in *MUTYH* [69]. Patients usually present with <100 colorectal polyps at an average age of disease diagnosis at around 50 years of age (which is similar to AFAP) and a high risk of CRC [69, 70]. The age of onset of polyposis has been shown to be significantly delayed for biallelic *MUTYH* carriers compared to *APC* mutation carriers [69]. MAP has been associated with malignancies of the duodenum, ovaries, urinary bladder and skin—occasionally resembling the phenotype of LS [71]. In a recent report describing extra-colonic disease, biallelic carriers are at high risk of urinary bladder and ovarian cancer, while there is some evidence that monoallelic carriers are at risk of gastric, hepatobiliary, endometrial and breast cancer [72]. No increased risk of other extra-colonic cancers associated with FAP was observed in this study [72].

### NTHL1-associated polyposis (NAP)

A recently described autosomal recessive polyposis condition has been named NAP (OMIM #616415). Patients have germline homozygous or compound heterozygous mutations in the base excision repair gene *NTHL1* [73]. Due to its recent discovery the clinical manifestation is not set, but it points towards an extended spectrum of cancer diagnosis in these patients; endometrial, duodenal, skin (basal cell carcinoma) and others [74]. Given such disease heterogeneity, Dutch researchers suggest it is a novel cancer syndrome. This is supported by Canadian researchers who also identified biallelic *NTHL1* mutations in a woman with multiple primary tumours [75].

### Polymerase proofreading-associated polyposis (PPAP)

PPAP is associated with mono- and biallelic mutations in the genes *POLE* and *POLD1* [76], both genes a part of the mismatch repair (MMR) pathway. PPAP is in an autosomal dominantly inherited CRC predisposition [77]. Variants in *POLE* and *POLD1* are known to increase the somatic mutation rate in tumours [78], thereby increasing the risk of tumour development. The somatic mutation landscape can display great diversity [79], which could be a reason for the differences observed in the location of primary tumours between patients. Both *POLE* and *POLD1* have been associated with an increased risk of endometrial cancer [76, 80]. *POLD1* has been associated with breast and brain tumours in addition to CRC and endometrial cancer [81]. Multi-tumour phenotypes such as colon/pancreas/ovaries/small intestine [82] and colon/ovarian/endometrial/brain [80] have been seen in *POLE* mutation carriers. In addition, *POLE* has been linked to an early onset cancer case raising the question whether this specific *POLE* mutation may confer a more severe phenotype than previously reported *POLE/POLD1* mutations [83].

## Conclusions

Genetic testing has rapidly grown in the last few years with the advancement of next-generation sequencing technologies. Targeted testing of all polyposis patients with a gene-panel can now be performed at reasonable cost such that targeted screening or prophylactic surgery can be offered to patients with a molecular diagnosis of polyposis.

In 2009 Half et al. [8] identified unresolved questions regarding FAP, one being that there are many FAP patients who do not get a molecular diagnosis. Since 2009, three additional genes have been associated with adenomatous polyposis, one being *NTHL1* which has been classified as NAP, and *POLE* and *POLD1* which has been classified as PPAP.

New novel mutations will continually be discovered in genes already associated with disease and new genes will be discovered that are associated with adenomatous polyposis. Exome sequencing has already been used to identify new candidate genes; *PIEZ01* and *ZSWiM7* [84], which are currently subject to further investigation.

A recent study has found that gene expression of *APC* was reduced in FAP patients without germline APC mutations [85]. An explanation may lie in differential epigenetic factors that contribute to the lack of gene expression in these patients, maybe more focus should be placed on understanding the role of epigenetics in polyposis syndromes.

There remain a high proportion of *APC* mutation negative patients even after extensive searches for new causative genes. The question remains, have we just missed them, or is it that these patients harbour rare alleles that await discovery. Diagnostics laboratories around the world are spending considerable amounts of time designing gene-panels to test for all adenomatous polyposis syndromes using new sequencing technology such that in the near future the number of *APC* mutation negative patients is expected to significantly decrease. Until then, re-testing “old” *APC* mutation negative patients for additional genes that have already been identified should be of special interest.
